# Hybrids of Gallic Acid@SiO_2_ and {Hyaluronic-Acid Counterpats}@SiO_2_ against Hydroxyl (^●^OH) Radicals Studied by EPR: A Comparative Study vs Their Antioxidant Hydrogen Atom Transfer Activity

**DOI:** 10.1021/acs.langmuir.4c02760

**Published:** 2024-12-07

**Authors:** Annita Theofanous, Yiannis Deligiannakis, Maria Louloudi

**Affiliations:** † Laboratory of Biomimetic Catalysis and Hybrid Materials, Department of Chemistry, University of Ioannina, Panepistimioupoli, Ioannina GR-45110, Greece; ‡ Laboratory of Physical Chemistry of Materials and Environment, Department of Physics, University of Ioannina, Panepistimioupoli, Ioannina GR-45110, Greece

## Abstract

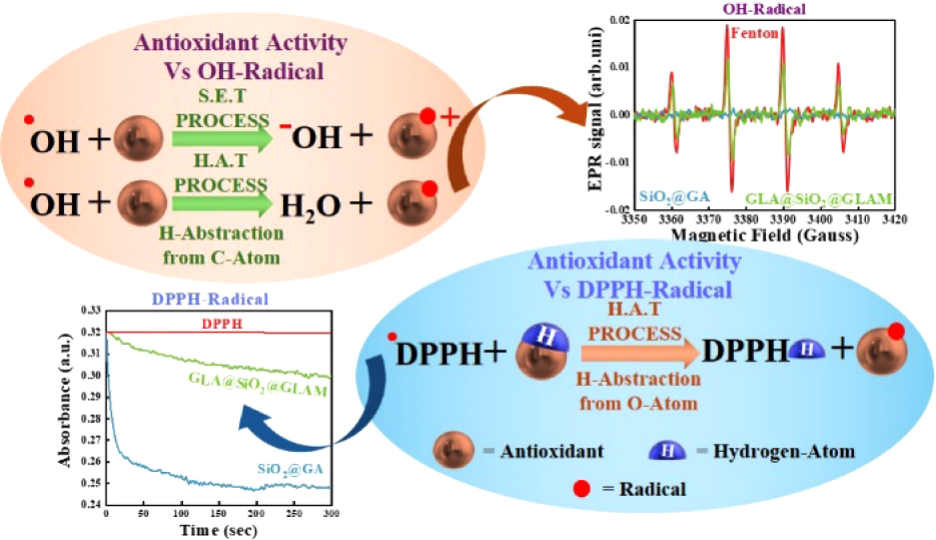

Hydrogen atom transfer (HAT) and single electron transfer (SET) are two fundamental pathways for antiradical/antioxidant processes; however, a systematic *in-tandem* operational evaluation of the same system is lacking. Herein, we present a comparative study of the HAT and SET processes applied to a library of well-characterized hybrid materials SiO_2_@GA, SiO_2_@GLA, SiO_2_@GLAM, and the doubly hybrid material {GLA@SiO_2_@GLAM}. Hydroxyl radicals (^•^OH), produced by a Fenton system, react via the single electron transfer (SET) pathway and hydrogen atom transfer, through oxygen- and carbon-atoms, respectively, while the stable-radical DPPH via the HAT pathway through oxygen-atoms. Electron paramagnetic resonance spectroscopy (EPR), eminently suited for *in situ* detection and quantification of free radicals, was used as a state-of-the-art tool to monitor ^•^OH using the spin-trapping-EPR method. We found that the SiO_2_@GA hybrid exhibited the highest SET ^•^OH-scavenging activity i.e., [2.7 mol of ^•^OH per mol of grafted GA]. Then, SiO_2_@GLA, SiO_2_@GLAM, and GLA@SiO_2_@GLAM can scavenge 1.2, 1.3, and 0.57 mol of ^•^OH per mol of anchored organic, respectively. The HAT efficiency for SiO_2_@GA was [2.0 mol of DPPH per mol of grafted GA], while SiO_2_@GLA, SiO_2_@GLAM, and GLA@SiO_2_@GLAM exhibited a HAT efficiency of 1.1 DPPH moles per mol of anchored organic. The data are analyzed based on the molecular structure of the organics and their −R–OH moieties. Accordingly, based on the present data we suggest that for hydroxyl (^•^OH) radicals, the mechanisms involved are SET from an oxygen atom and HAT from a carbon atom. In contrast, for DPPH radicals, the HAT mechanism is exclusively operating and involves hydrogen atom abstraction from OH groups.

## 1 Introduction

Highly reactive free radicals and reactive oxygen species (ROS) substantially participate in a multitude of pathophysiological processes.^1^ Free radicals are postulated to function as key regulators in modulating diverse physiological cellular processes.^2^ Elevated concentrations of reactive oxygen species (ROS) and reactive nitrogen species (RNS) possess the capacity to engage in interactions with fundamental biomolecules. Such interactions can lead to structural and functional alterations.^3^ Reactive oxygen species (ROS) are conventionally produced within cellular environments; however, living organisms are equipped with a repertoire of both enzymatic and nonenzymatic antioxidant mechanisms to govern and alleviate the deleterious impact of these species.^4^ Oxidative stress is delineated by a state of disequilibrium between the production of reactive oxygen species (ROS) and the cellular ability to orchestrate a proficient antioxidant defense mechanism.^5^

Amidst the plethora of reactive oxygen species (ROS), the hydroxyl radical (^•^OH) assumes a pivotal role as a crucial intermediate in “green oxidations,″ primarily due to its significantly heightened standard redox potential of 2.73 V concerning the reversible hydrogen electrode (RHE).^6^ The hydroxyl radical (^•^OH) stands as an extraordinarily reactive oxidizing agent, holding paramount significance across diverse domains including chemistry, biology, medicine, and the fields of atmospheric and environmental science.^7^ It is an exceptionally reactive molecular species endowed with the capacity to inflict substantial damage upon the host cell through oxidative alterations of amino acids.^8^

**Scheme 1:**
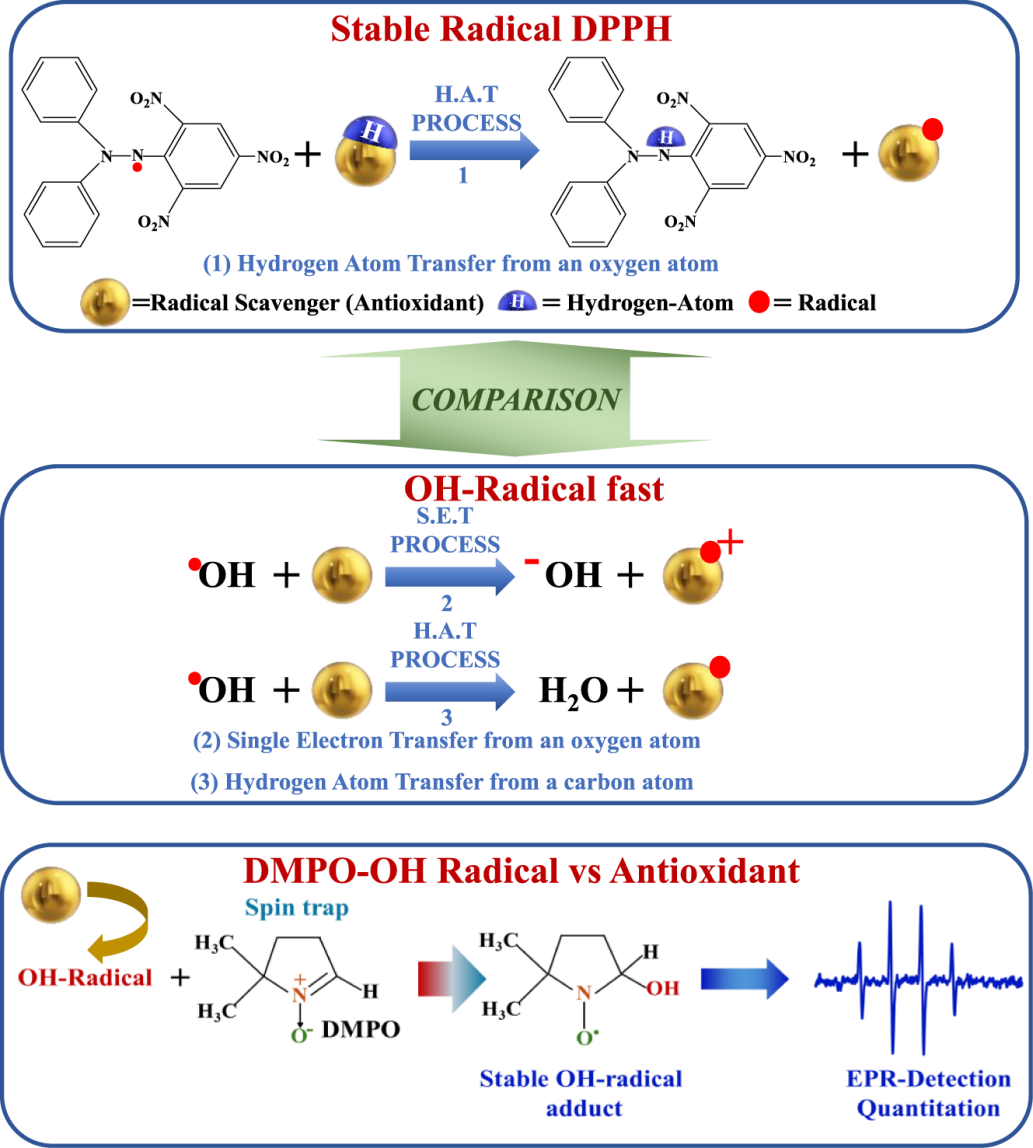
Schematic Representation of the Three Possible Reaction Paths for an Antioxidant Radical-Scavenging Agent In the case of the stable radical (DPPH), hydrogen atom transfer may operate from an oxygen atom. In the case of fast OH-radicals, a single-electron transfer is the prevalent mechanism, and the HAT mechanism may operate with the abstraction of a hydrogen atom from a carbon atom. The fast OH-radicals can be studied in situ by EPR spectroscopy via a rapid spin trapping technique using DMPO.

Nevertheless, the effort to detect OH radicals faces significant challenges due to their extremely short half-life and unrestrained reactivity.^9,10^ Electron paramagnetic resonance spectroscopy (EPR) serves as a dedicated analytical method^11^ meticulously crafted for the precise detection and quantification of free radicals.^12^ This approach involves a chemical interaction between a free radical and a diamagnetic molecule, commonly referred to as a spin-trap,^13,14^ leading to the formation of a radical species characterized by an extended half-life (see Scheme 1), denoted as a spin adduct.^13,14^ This technique offers unparalleled accuracy in the identification of free radicals.^12,15^

To form ^•^OH *on demand* for research reasons, the Fenton reaction is employed. In the course of the Fenton reaction, ferrous ions (Fe^(II)^) react with hydrogen peroxide (H_2_O_2_), leading to the creation of hydroxyl radicals (^•^OH).^16^ Iron stands as the most frequently utilized metal in Fenton chemistry. Within this framework, the reduction of hydrogen peroxide (H_2_O_2_) by ferrous ions (Fe^II^) culminates in the generation of ferric ions (Fe^III^) and hydroxyl radicals (^•^OH).^6^ To attain detectability, the hydroxyl radical necessitates initial entrapment by 5,5-dimethyl-1-pyrroline *N*-oxide (DMPO), culminating in the creation of a relatively stable paramagnetic adduct acknowledged as DMPO–OH^•^ (see Scheme 1).^16,17^

The quantification of antioxidant capacity in plant extracts is frequently determined by assessing the scavenging activity against hydroxyl radicals generated through the Fenton reaction.^18^ Antioxidants, characterized as radical-abating moieties, engender profound scientific and economic interest across various domains, including but not limited to human health, food science, and polymer research.^19−21^

When a substance, present in limited concentrations, demonstrates the capacity to impede or decelerate the process of autoxidation exhibited by free radicals, it is categorized as an antioxidant. An antioxidant, when found in diminished quantities, faces challenges in effectively outcompeting highly reactive hydroxyl (^•^OH) and peroxyl (PO^•^) radicals, owing to the latter’s pronounced reactivity toward organic molecular species.^5^ In the context of the interaction between an antioxidant and a free radical, it is essential to recognize that the antioxidant serves as the oxidizing agent and undergoes oxidation, whereas the free radical undergoes reduction. Consequently, the antioxidant plays a crucial role in safeguarding the neutral substrate from the deleterious effects of the free radical (see Scheme 1).^14^

In the past decade, the methodical incorporation of natural antioxidants into material matrices has emerged as a sophisticated technological approach aimed at mitigating inherent constraints associated with these antioxidants.^21^ An approach to enhance the attributes of antioxidants involves the incorporation of diminutive antioxidant molecules onto nanoparticles. The nanomaterial supports chosen for the immobilization of antioxidants necessitate characteristics of nontoxicity, cost-effectiveness, and functional versatility.^5^ This approach enables the emulation of natural antioxidant systems at the nanoscale level.^22^

To fulfill this objective, Li et al. have achieved the successful fabrication of hollow short linear glucan (SLG)@gum Arabic (GA) nanospheres,^23^ and Sudha et al. employed silver nanoparticles (AgNPs) on *Lippia nodiflora*.^24^ The research of Medhe et al. suggests that 3,6-dihydroxyflavone incorporating gold nanoparticles demonstrates enhanced antioxidant properties compared to their unmodified counterparts,^25^ while Ren et al. synthesized gallic acid-quaternized chitosan, and caffeic acid-quaternized chitosan.^26^ On the other hand, Li et al. orchestrated the conception and successful synthesis of a series of novel chitosan quaternary ammonium derivatives, incorporating pyridine or amino-pyridine moieties.^27^ Magomedova et al. synthesized a series of novel cationic starch derivatives, incorporating 1,2,3-triazole functionalities, utilizing “click” reactions and N-alkylation techniques.^28^ Gold nanoparticle (AuNP)-modified cerium dioxide (CeO_2_) nanomaterials with three distinct morphologies were synthesized by Fa et al.^29^ All of the aforementioned materials were evaluated for their efficacy in neutralizing hydroxyl (^•^OH) radicals, which were generated through the Fenton reaction. UV–vis spectroscopy was employed for the analysis of all of the samples.

The investigation into the antioxidant activity of nanoceria was conducted via electron paramagnetic resonance (EPR) spectroscopy, employing 5,5-dimethyl-1-pyrroline N-oxide (DMPO) as a spin trap.^30^ Lipid-coated polydopamine nanoparticles (L-PDNPs) are suggested by Battaglini et al. as multifunctional agents serving dual roles as antioxidants.^31^ Qui et al. assessed the antioxidative efficacy of materials derived from graphene by evaluating their capacity to scavenge hydroxyl (^•^OH) radicals.^32^

Another group of nanoparticles, bimetallic Ag/Cu and Cu/Zn nanoparticles, was synthesized by Merugu et al. The investigation into antioxidant capacity involved various methods, including the scavenging of hydroxyl (^•^OH) radicals. In pursuit of this objective, high-performance liquid chromatography (HPLC) spectrophotometry was employed.^33^ Furthermore, Sharma et al. synthesized Au/Ag bimetallic nanoparticles (BMNPs) through a one-step process. The underpinning principle of this assay involves the formation of a chromophoric compound resulting from the oxidative degradation of 2-deoxyribose mediated by hydroxyl radicals.^34^

Morin-modified mesoporous silica nanoparticles (AMSNPs-MOR) were synthesized by Arriagada et al.^35^ Sandhya and collaborators employed a green synthesis approach to fabricate iron oxide nanoparticles utilizing an extract derived from *B. flabellifer*.^36^ Fourteen innovative derivatives of chitosan oligosaccharides (COS), functionalized with phenolic acids, were successfully synthesized by Sun et al.^37^ Pasanphan et al. synthesized an innovative antioxidant (chitosan–GA) resulting from the conjugation of the biopolymer chitosan with gallic acid.^38^ All of the materials above were assessed for their efficacy in scavenging hydroxyl (^•^OH) radicals, which were produced via the Fenton reaction.

**Scheme 2:**
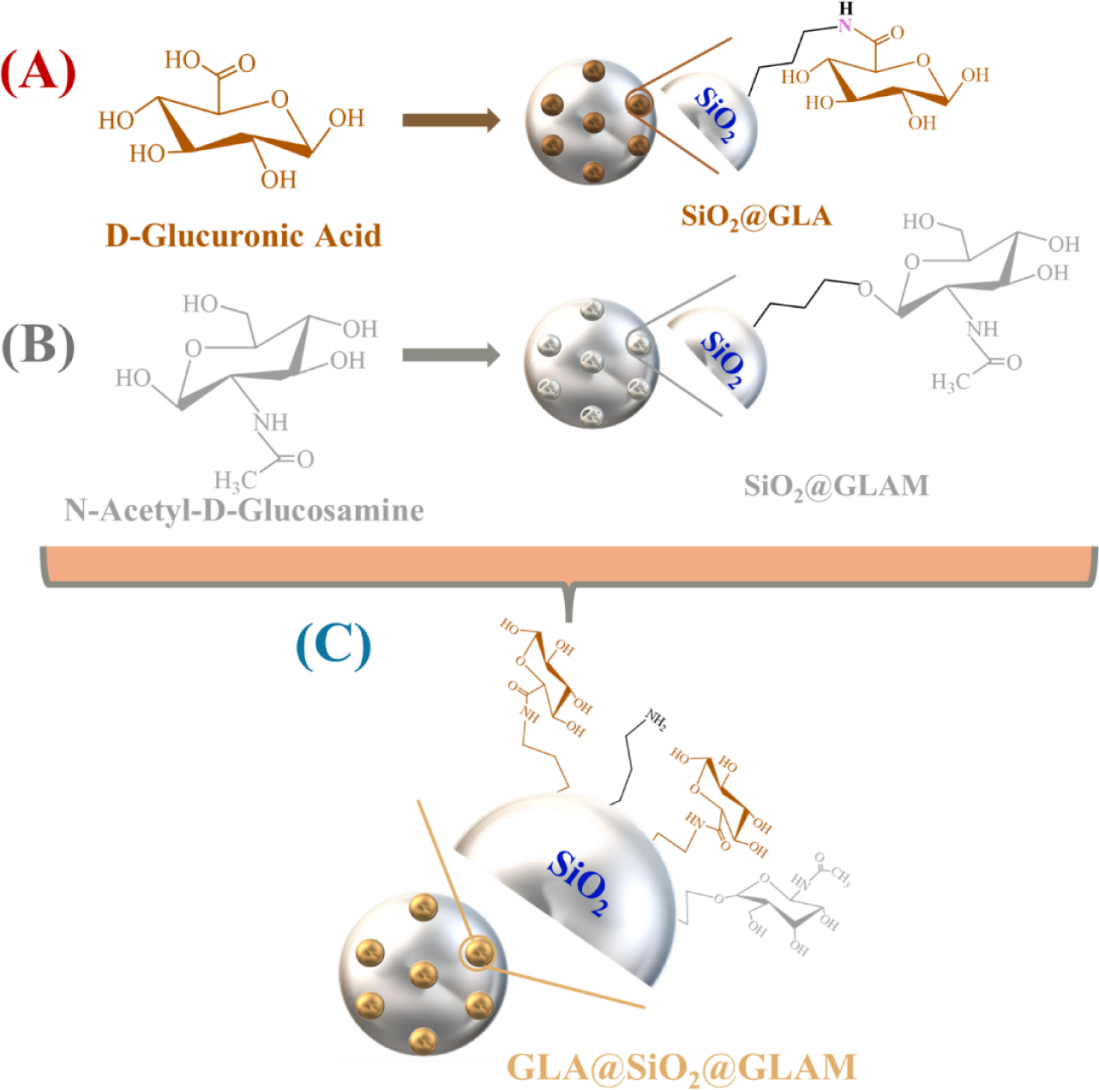
Structural Representation of (A) {SiO_2_@GLA}, (B) {SiO_2_@GLAM}, and (C) the Doubly Grafted {GLA@SiO_2_@GLAM} Nanohybrids

In recent research, we have shown that hybrid-antioxidant nanomaterials can be engineered employing silica as the support matrix and incorporating gallic acid.^39^ Subsequently, the monomeric constituents of hyaluronic acid, namely d-glucuronic acid and *N*-acetyl-d-glucosamine, were immobilized onto the SiO_2_ nano matric, serving as antioxidant moieties (Scheme 2).^21^ Following the successful synthesis of the materials, an investigation into their antioxidant activity against DPPH radicals was conducted employing the hydrogen atom transfer (HAT) mechanism.^21^

**Scheme 3:**
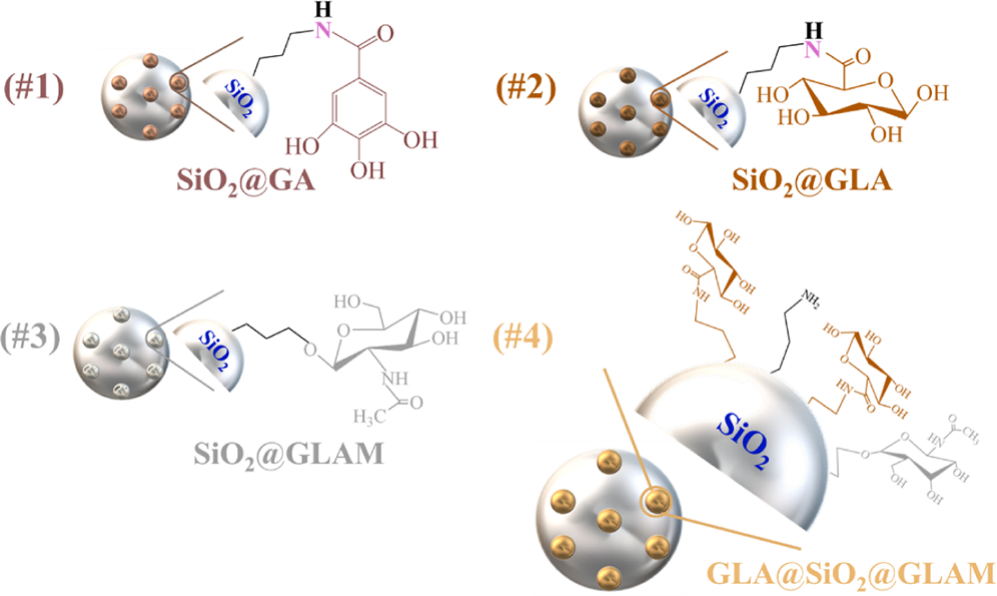
Illustration of the Hybrid Materials’ Molecular Structures, Code Names, and Numerical Identifiers

Despite the widely anticipated primary importance of the ^•^OH-scavenging and HAT-antioxidant mechanisms, a direct comparison of the same-scavenger toward these two processes is rather scarce.^40−42^ Moreover, such a comparison for hybrid antioxidants is lacking in the literature. This entails the need for such a study, i.e., to understand the similarities/differences in reaction pathways and efficiencies of the two processes (Scheme 1). Additionally, Scheme 3 and Table 1 present the molecular structures and corresponding codename of each hybrid material, along with the numbering assigned to each compound.

**Table 1:** Code Designations of the Hybrid Materials

Hybrid Material	Code Name
SiO_2_@GA	#1
SiO_2_@GLA	#2
SiO_2_@GLAM	#3
GLA@SiO_2_@GLAM	#4

In this context, herein, we present a study that aims (i) to compare directly the same radical-scavenging hybrids #1, #2, #3, and #4 against hydroxyl (^•^OH) radicals; (ii) to compare their OH-scavenging vs the HAT-scavenging efficiency using 2,2-diphenyl-1-picrylhydrazyl (DPPH) radicals as HAT-probes; (iii) to peer into the relative similarities and differences with a focus on single electron transfer (SET) vs hydrogen atom transfer (HAT) (see Scheme 1). It is underlined that a computational/theoretical analysis of these systems was, at present, out of the present scope. We focus on the experimental data and their comparative analysis.

Regarding the ability to scavenge hydroxyl (^•^OH) radicals, hybrid material no. 1 exhibited the highest efficacy among all the materials tested. The ranking of hybrid materials from most to least effective is as follows: #1 > #2 > #3 > #4. Conversely, concerning the ability to scavenge DPPH radicals, the materials are ranked as follows: #1 > #4 > #2 > #3. The doubly grafted hybrid material is less effective in binding ^•^OH than DPPH radicals due to the steric obstructions caused by the GLA and GLAM entities grafted on the silica surface.

## 2 Materials and Methods

### 2.1 Chemicals and Solvents

Ferrous(II) sulfate heptahydrate (FeSO_4_·7H_2_O, >99.5%) was obtained from Fluka. Hydrogen peroxide solution (H_2_O_2_ 30 wt %) and 5,5-dimethyl-pyrroline N-oxide (DMPO) were obtained from Sigma-Aldrich. 2,2-Diphenyl-1-picrylhydrazyl (DPPH) was also obtained from Sigma-Aldrich. Water for chromatography (LC-MS grade), 2.5 L, was obtained from Merck. d-Glucuronic acid (GLA, > 99%) and *N*-acetyl-d-glucosamine (GLAM, >99%) were obtained from Sigma-Aldrich. Silica gel 60 (0.040–0.063 mm) for column chromatography was obtained from Merck. Gallic acid (GA) 1 kg was obtained from Sigma-Aldrich.

### 2.2 Synthesis of Hybrid Materials

The synthesis methodology for the hybrid materials was extensively documented and reported in our preceding publication. The key steps involve anchoring the organics GA, GLA, and GLAM onto the silica surface via the sol–gel methodology, which proceeds through transient silane-precursor formation. Their hydrolysis and condensation on surficial silica sites result in the synthesis of the hybrid materials #1, #2, and #3.^21,39^ For the synthesis of the doubly grafted hybrid material, both d-glucuronic acid (GLA) and *N*-acetyl-d-glucosamine (GLAM) are grafted onto the same silica matrix in a molar ratio of [GLA: GLAM] [2:1].^21^

### 2.3 Instrumentation

#### 2.3.1 Electron Paramagnetic Resonance (EPR) Spectroscopy

Electron paramagnetic resonance (EPR) measurements were conducted by employing a continuous-wave Bruker ER200D spectrometer, complemented by an Agilent 5310A frequency counter. The spectrometer was operated through custom-designed software implemented in LabView. The parameters for acquiring EPR spectra were set as follows: modulation amplitude of 10 G peak-to-peak (Gpp), modulation frequency of 100 kHz, and microwave power of 20 mW. The spectra were recorded under ambient room temperature conditions.

### 2.4 Evaluation of the OH-Radical Scavenging Capacity (^•^OH-RSC)

The assessment of the OH-radical scavenging capacity for all hybrid materials and their respective monomers was conducted utilizing electron paramagnetic resonance (EPR) spectroscopy. The generation of hydroxyl radicals (^•^OH) is achieved via the Fenton reaction.^43^ Hydroxyl radicals are generated within an aqueous solution comprising ferrous ions (Fe^(II)^) and hydrogen peroxide (H_2_O_2_). To initiate the production of hydroxyl radicals (^•^OH), hydrogen peroxide (H_2_O_2_) at *C* = 50 μΜ interacts with Fe^II^ (FeSO_4_)·7H_2_O (1 μM) in water for chromatography (LC-MS grade) at pH = 6.7.

The formed OH-radicals were trapped using 6.67 mM of DMPO (5,5-dimethyl-1-pyrroline *N*-oxide),^44^ according to Scheme 1. Using a total reaction volume of 3 mL, to measure the EPR signals of the DMPO–OH prepared stock suspension, 20 μL was dispensed into glass capillaries and subsequently subjected to in situ irradiation within the electron paramagnetic resonance (EPR) cavity. According to Lloyd et al.,^45^ the spin-trapping process of OH by DMPO in the Fenton system proceeds *via* a rapid, selective formation of the DMPO–OH^•^ adduct that is characterized by the characteristic quadruplet with a 1:2:2:1 intensity pattern, see Scheme 1.^45^

As a control, we have verified that our SiO_2_ particles when interacting with H_2_O_2_ of the Fenton reaction, at the concentration used in our antioxidant experiment, produce insignificant concentrations of OH-radicals i.e., of the order of 10^–2^ μM ΟΗ-radicals. This is negligible, compared with the OH produced by the Fenton reaction, >4 μM (see below).

#### 2.4.1 OH-Radical Monitoring Methodology

OH-radical generation via the Fenton Fe/H_2_O_2_ system is well-known^16,17,43^ and involves many intermediate steps that occur through Fe^2+^/Fe^3+^ redox-cycling^16^ and H_2_O_2_/OH-radical transformations. Herein, to avoid unnecessary complications, we focus exclusively on the formation of OH-radicals. Thus, before the addition of any radical-scavenger, the key reactants [H_2_O_2_ and iron] were allowed to react for 30 s at 25 °C, i.e., to maximize the OH-radical formation. Detailed screening experiments using EPR monitoring of the DMPO–OH^•^ radical adducts showed that at 30 s, the formed ^•^OH population attained a steady state of 4.15 μM ΟΗ-radicals. When no antioxidant was added, the DMPO–OH^•^ population remained steady for 15–20 min. Thus, using this time-window, the addition of the antioxidants at 30 s provided reproducible, quantitative trends in the ^•^OH populations. Another crucial step of pertinence examined was the sequence of addition of the antioxidant/DMPO-spin trap. It is underlined that the correct sequence is as anticipated in Scheme 1, lower part: [1] formation of the OH-radicals by the Fe/H_2_O_2_ Fenton system → 30 s evolution, [2] addition of the antioxidant → allow time for the antioxidant to act, and [3] add DMPO to monitor the OH-radicals remaining. Quantitative analysis of hydroxyl radicals (^●^OH) was performed utilizing DPPH (1,1-diphenyl-2-picrylhydrazyl) as a spin standard,^43,46^ alongside the calculation of the double integral of the electron paramagnetic resonance (EPR) signals.^44^ The total OH-radicals, formed from Fenton, OH_total_, and the nonscavenged radicals after adding the antioxidant materials i.e., {#1, #2, #3, or #4} were quantified by EPR. Finally, the scavenged OH radicals were estimated accordingly.
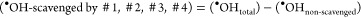
the ratio *n*= (moles ^•^OH-scavenged)/(moles GA, GLA, GLAM) was estimated using the moles of GA, GLA, and GLAM per mass of {#1, #2, #3, #4}, determined by TGA analysis^21^ (see Table 1).

## 3 Results and Discussion

### 3.1 ^•^OH-Scavenging Activity: EPR Study

**Figure 1:**
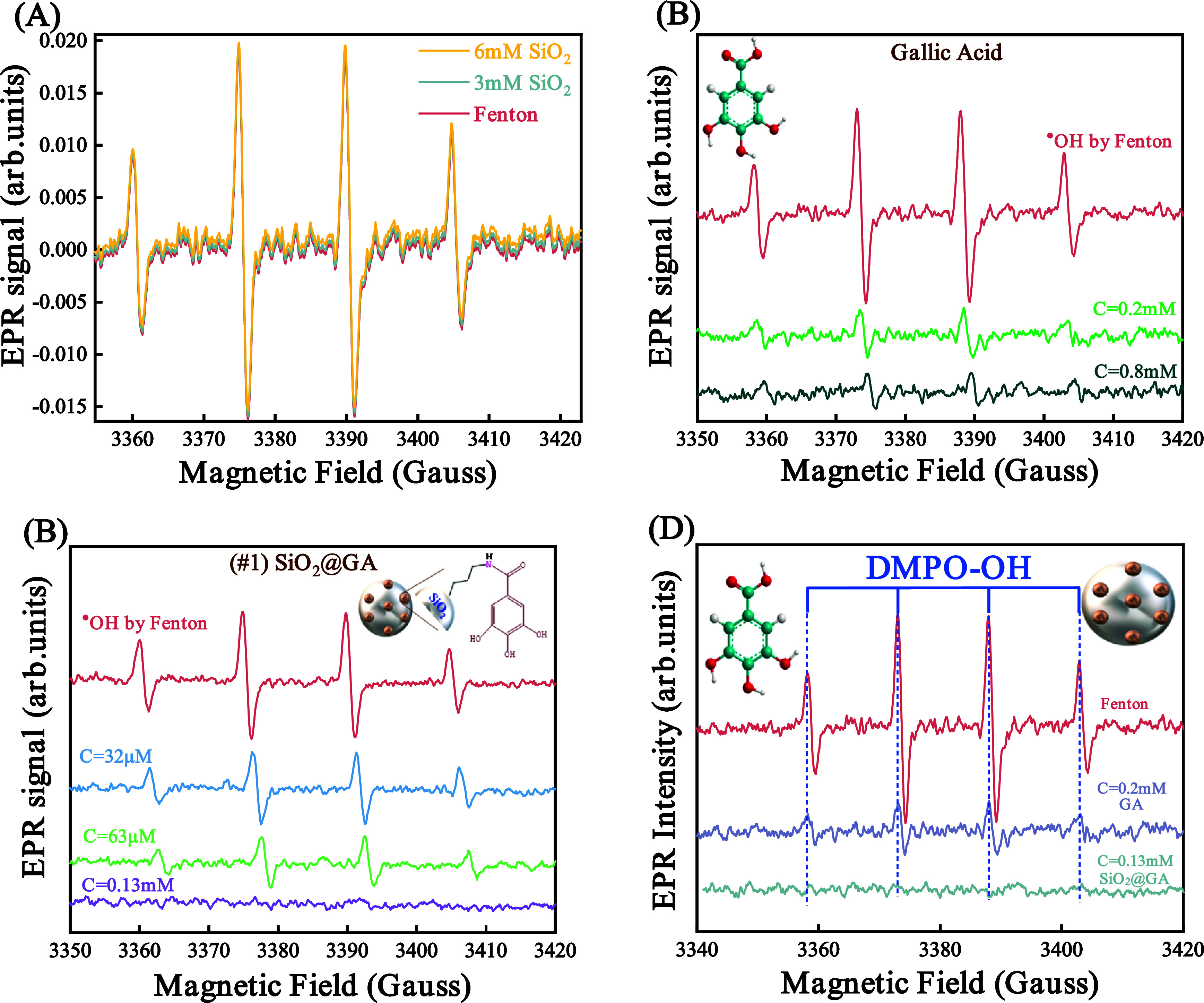
EPR spectra demonstrating the reduction in the concentration of OH radicals within the solution in the presence of (A) SiO_2_, (B) gallic acid (GA), and (C) SiO_2_@GA. The red line presents the control test without the addition of antioxidant species. (D) The comparison of EPR spectra reveals that hybrid material #1 exhibits superior efficacy in neutralizing hydroxyl (^•^OH) radicals compared to free, nongrafted GA.

The solution containing DMPO–OH^•^ radicals exhibits the characteristic four-line EPR spectrum characterized by a 1:2:2:1 intensity peak pattern (Figure 1).^47−50^ The introduction of antioxidants leads to the quenching of hydroxyl (^•^OH) radicals, as evidenced by a decrease in the peak intensity observed in EPR spectra. No other/secondary radicals were detected in the present experiments.

To assess the antioxidant efficacy of gallic acid and the SiO_2_@GA hybrid, the experimental protocol outlined in section 2.4 was executed. Gallic acid was introduced into Fenton’s solution at concentrations of *C* = 0.2 mM and *C* = 0.8 mM of gallic acid, while material #1 was added at concentrations of *C* = 32 μM, *C* = 62 μM, and *C* = 0.13 μM of material #1. Figure 1A–C below depicts the schematic representations of DMPO–OH^•^ radicals in the presence of gallic acid (GA), and material #1, vs the control experiment.

The data in Figure 1 reveal that material #1 was able to achieve comparative ^•^OH-scavenging to pure gallic acid, at much lower concentrations of grafted-GA. Figure 1D allows direct visualization of this effect, i.e., 0.2 mM GA performs less efficiently than 0.13 mM material #1. As a control, SiO_2_ alone had a negligible effect on the ^•^OH-EPR signals, see the trace in Figure 1A.

Hence, the data in Figure 1 reveal that the covalent attachment of gallic acid onto the silica surface confers enhanced antioxidant activity to the hybrid material against hydroxyl (^•^OH) radicals. In summary, 1 g of GA grafted onto SiO_2_ effectively scavenges 16.1 mmol of hydroxyl (^•^OH) radicals, whereas 1 g of pure gallic acid (GA) scavenges 13.2 mmol of OH radicals.

**Figure 2:**
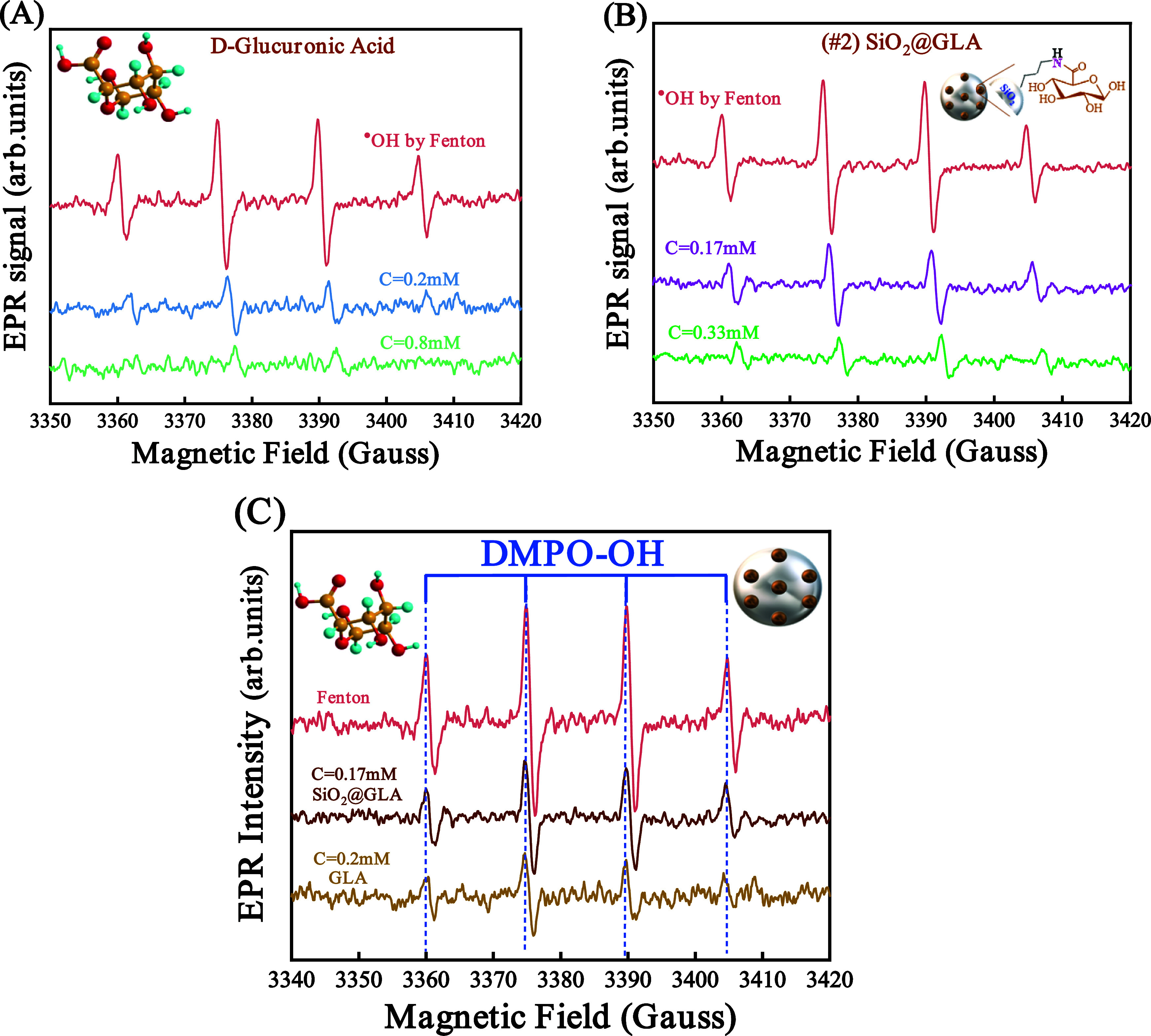
EPR spectra illustrate the decrease in the concentration of hydroxyl (^•^OH) radicals within the solution upon the introduction of (A) d-glucuronic acid (GLA) and (B) SiO_2_@GLA. The red line presents the control test without the addition of antioxidants. (C) The comparative analysis of electron paramagnetic resonance (EPR) spectra indicates that hybrid material #2 demonstrates slightly lower effectiveness in scavenging hydroxyl (^•^OH) radicals than its pristine GLA molecule.

To evaluate the antioxidant potency of d-glucuronic acid and the corresponding SiO_2_@GLA hybrid, we followed the experimental protocol delineated in section 2.4. d-Glucuronic acid was introduced into Fenton’s solution at concentrations of *C* = 0.2 mM and *C* = 0.8 mM, while the hybrid material #2 was incorporated at concentrations of *C* = 0.17 mM and *C* = 0.33 mM. Figure 2A,B illustrates schematic representations of DMPO–OH^•^ radicals juxtaposed with d-glucuronic acid (GLA) and material #2, respectively.

In the comparative evaluation between hybrid material #2 and d-glucuronic acid, a contrasting pattern emerges compared to that observed with [gallic acid and material #1]. Specifically, the covalent attachment of glucuronic acid to the silica surface leads to a slight decrease in the capacity to scavenge ^•^OH compared to the pure glucuronic acid molecule. The quantitative assessment of the two antioxidants revealed that 1 g of d-glucuronic acid can effectively scavenge 7.4 mmol of ^•^OH, whereas 1 g of GLA covalently attached to SiO_2_ demonstrates the capability to scavenge 6.2 mmol of ^•^OH radicals.

**Figure 3:**
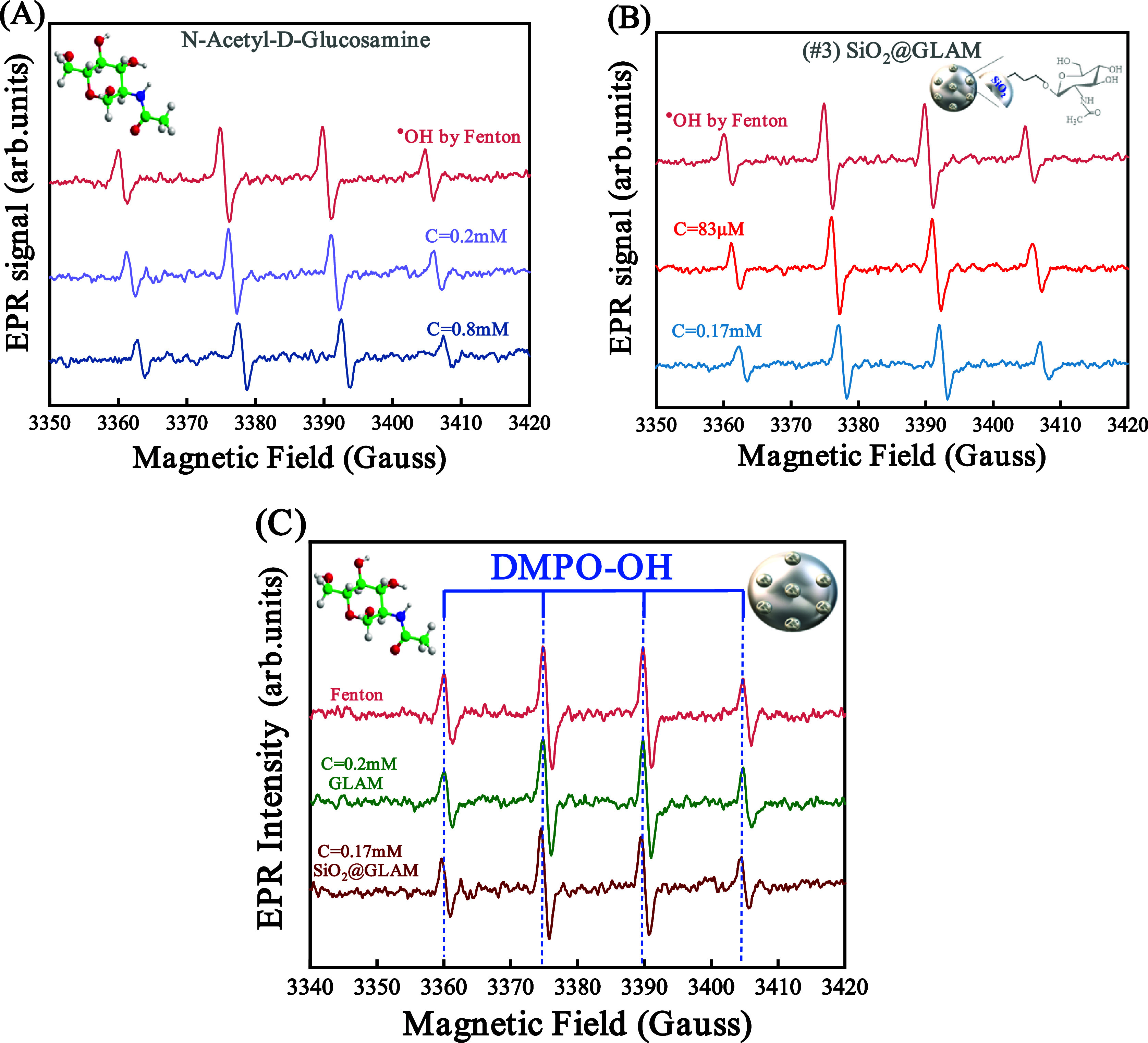
EPR spectra elucidate the reduction in the concentration of hydroxyl (^•^OH) radicals within the solution after the introduction of (A) *N*-acetyl-d-glucosamine (GLAM) and (B) SiO_2_@GLAM. In both figures, the spectrum on the top presents the control test without the addition of antioxidants. (C) The comparative examination of electron paramagnetic resonance (EPR) spectra reveals that the *N*-acetyl-d-glucosamine exhibits reduced efficacy in scavenging hydroxyl (^•^OH) radicals compared to its hybrid material #3.

Subsequently, antioxidant assessment of the *N*-acetyl-d-glucosamine hyaluronic acid monomer and the corresponding SiO_2_@GLAM hybrid material was conducted. Like the preceding experiments, GLAM was introduced into the Fenton reaction solution at concentrations *C* = 0.2 mM and *C* = 0.8 mM, whereas the hybrid material #3 was incorporated into the Fenton reaction at concentrations *C* = 83 μM and *C* = 0.17 mM. Figure 3A,B below illustrates the electron paramagnetic resonance (EPR) spectra of DMPO–OH^•^ after the addition of various concentrations of GLAM (A) and material #3 (B).

Quantitative calculations demonstrate a consistent trend akin to that observed in GA and material #1, wherein the hybrid material displays enhanced antioxidant efficacy against OH radicals compared to its nongrafted form. Notably, 1 g of GLAM grafted on the SiO_2_ surface exhibits the capacity to scavenge 5.7 mmol of OH radicals, while an equivalent amount of GLAM scavenge 2.7 mmol of hydroxyl (^•^OH) radical. Figure 3C presents a comparative electron-paramagnetic resonance (EPR) spectrum for the two materials.

**Figure 4:**
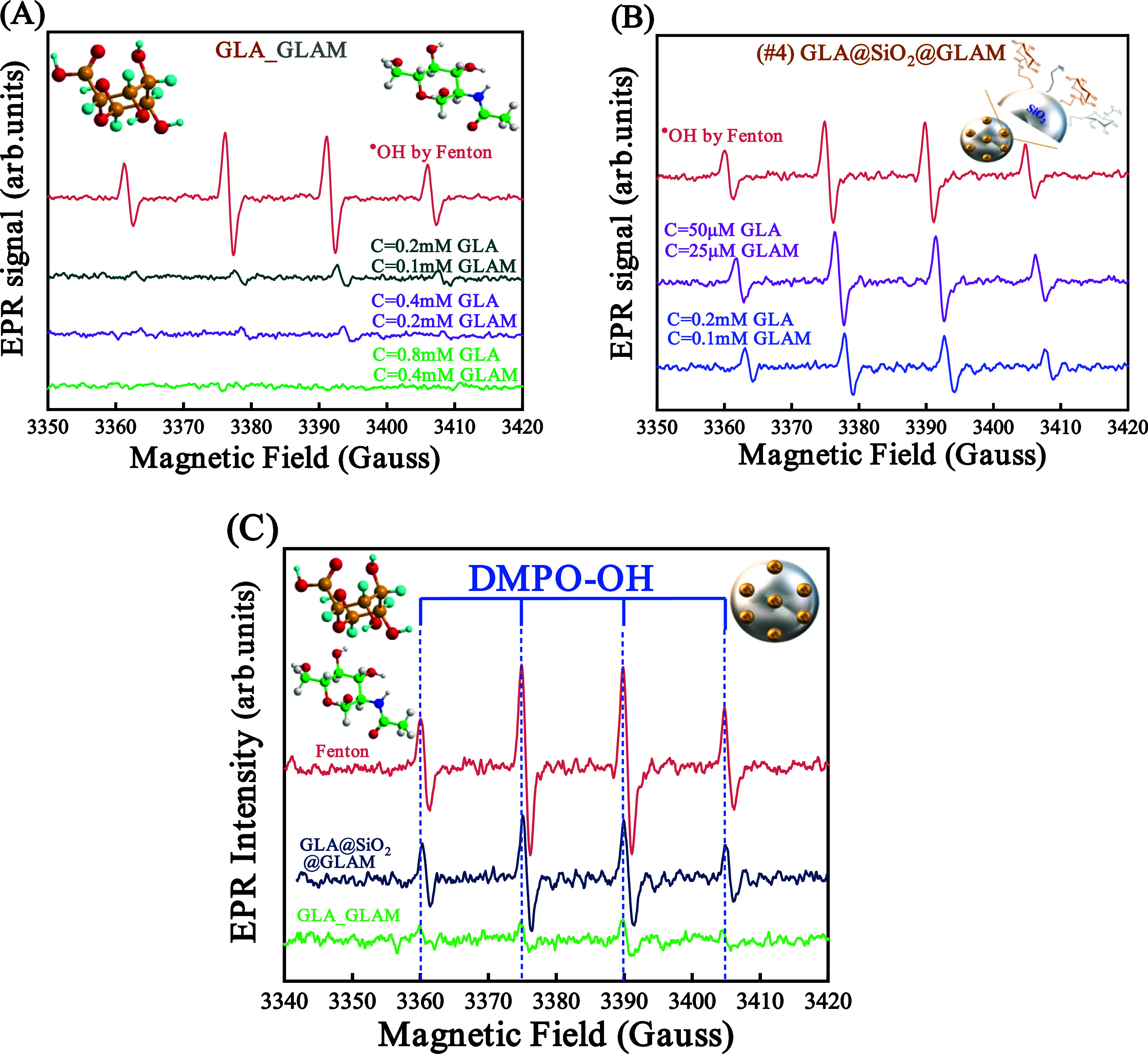
Electron paramagnetic resonance (EPR) spectra elucidate the decrement in the concentration of hydroxyl (^•^OH) radicals within the solution following the introduction of (A) simple mixture GLA–GLAM (2:1), and (B) doubly grafted hybrid material GLA@SiO_2_@GLA. The red line represents the control test without the addition of antioxidant species. (C) The comparative EPR analysis of the doubly grafted hybrid material #4 (molecular ratio 2:1) in scavenging hydroxyl (^•^OH) radicals vs the simple mixture counterpart, [GLA–GLAM] [2:1].

Ultimately, the efficacy of the simple mixing of GLA–GLAM (2:1) and the doubly hybrid GLA@SiO_2_@GLAM (2:1) material against ^•^OH was assessed through EPR spectroscopy, considering that the natural hyaluronic acid contains GLA: GLAM monomers in a ratio equal to 2:1.^51,52^ Initially, Fenton’s solution is prepared by combining Fe^(II)^ and H_2_O_2_, followed by a 30 s incubation period for the reaction to occur. Subsequently, *C* = 0.2 mM GLA, *C* = 0.1 mM GLAM, *C* = 0.4 mM GLA, *C* = 0.2 mM GLAM, and *C* = 0.8 mM GLA, *C* = 0.4 mM GLAM are promptly introduced (Figure 4A). Each alteration in the concentrations of the GLA–GLAM material necessitates the preparation of a new Fenton solution. The identical experimental protocol was replicated to assess the antioxidant efficacy of the doubly grafted hybrid material #4; however, the concentrations utilized were *C* = 50 μM GLA, *C* = 25 μM GLAM, *C* = 0.2 mM GLA, and *C* = 0.1 mM GLAM, Figure 4B.

Figure 4C illustrates that the covalent attachment of hyaluronic acid monomers, specifically d-glucuronic acid, and *N*-acetyl-d-glucosamine, onto the silica surface in a stoichiometric ratio of 2:1 leads to a decrease in the capacity of the [GLA–GLAM] mixture to scavenge OH radicals. 1 g of GLA–GLAM of the simple mixture material [GLA–GLAM] (2:1) can scavenge 4.7 mmol of ^•^OH. On the contrary, 1 g of GLA and GLAM attached to the silica surface can neutralize 2.1 mmol of ^•^OH. As illustrated in Figure 4A–C below, GLA–GLAM monomers demonstrate superior antioxidant activity compared to the doubly immobilized hybrid material #4.

Overall, Table 2 summarizes the present findings of hydroxyl (^•^OH) radicals scavenged per gram of antioxidant entity. According to Table 2, the hierarchical order of the ^•^OH scavenging efficiency is
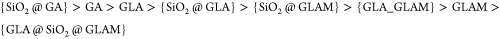


**Table 2:** ^•^OH Quenching Capacity (mmol) Per Gram of Antioxidant Material

Material	^•^OH scavenging (mmol per gram of material) (±0.01)
GA	13.2
#1: SiO_2_@GA	16.1
GLA	7.4
#2: SiO_2_@GLA	6.2
GLAM	2.7
#3: SiO_2_@GLAM	5.7
{GLA_GLAM}^a^[2:1]	4.7
#4: {GLA@SiO_2_@GLAM}^a^[2:1]	2.1

a The molar ratio [2:1] refers to [GLA: GLAM] grafted molecules on SiO_2_based on TGA analysis.

To increase precision and minimize error, each experiment was conducted in quadruplicate to ensure the reproducibility of the results.

### 3.2 Comparison of the Materials’ Capacity to Scavenge ^**•**^OH and DPPH Radicals

In our prior research on the HAT process by the #2, #3, and #4 hybrid materials,^21,39^ we have shown that the hybrid materials as well as the free molecules GLA and GLAM exhibited different antioxidant activity profiles toward DPPH radicals. Table 3 provides a summary of the material’s capacity to scavenge both ^•^OH and DPPH radicals.

**Table 3:** ^•^OH Quenching Capacity (mmol) Per Gram of Antioxidant Material and DPPH Quenching Capacity (mmol) Per Gram of Antioxidant Material

Materials	^•^OH scavenging (mmol/g of material) (±0.01)	Total DPPH scavenging (mmol/g) (±0.1)	^•^OH scavenging (mol OH/mol of antioxidant moiety) (±0.01)	DPPH scavenging via HATn_fast_ (±0.2)
GA	13.2	8.8	2.24	1.6
#1	16.1	11.7	2.70	2.0
GLA	7.3	0	1.44	0
#2	6.2	5.7	1.22	1.1
GLAM	2.7	0	0.6	0
#3	5.7	0.9	1.27	0.2
{GLA_GLAM}[2:1]	4.7	0	1.05	0
#4	2.1	5.7	0.57	1.1

The observed divergent antioxidant responses of our materials concerning ^•^OH and DPPH radicals imply the engagement of different underlying mechanisms to combat each type of radical.^53,54^ Gallic acid, being a well-known phenolic compound ubiquitously present in nature, as well as the hybrid material #1, appears to exhibit very high antioxidant capacity toward both DPPH radicals (i.e., via HAT) and hydroxyl (^•^OH) radicals in an aqueous medium. GA exerts its antioxidant activity primarily through two mechanisms: hydrogen atom transfer (HAT) and single electron transfer (SET), thereby mitigating oxidative stress and contributing to its role as a natural antioxidant.^55,56^ In an aqueous solution, GA scavenges hydroxyl radicals (^•^OH) predominantly through the single electron transfer (SET) mechanism, and the radicals are neutralized through a redox reaction;^57^ in this process, the GA donates an electron to the hydroxyl radicals, resulting in their reduction and cease to exist as free radicals, while GA is oxidized toward a cationic radical (GA^•+^) (Reaction 1).
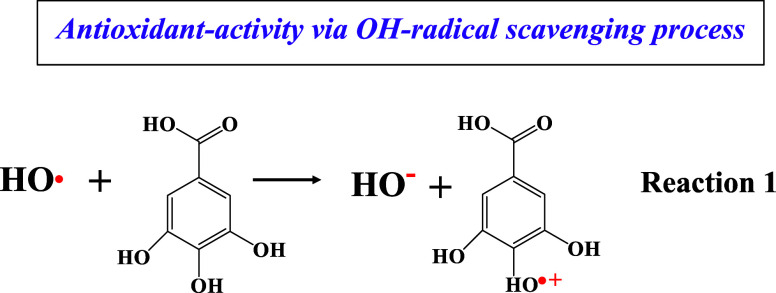


Regarding the neutralization of DPPH radicals in nonaqueous media, GA operates via the hydrogen atom transfer (HAT) mechanism, as extensively analyzed in previous studies, through the same sites (Reaction 2),^39^ explaining in some extent the analogous behavior in terms of antioxidant activity against both ^•^OH and DPPH radicals.
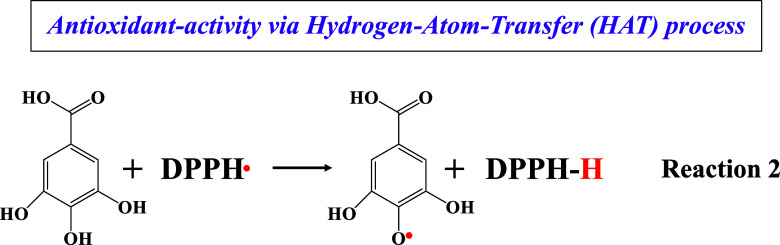


Concerning the hybrid material #2 and the free GLA, it appears that both exhibit high antioxidant activity vs hydroxyl (^•^OH) radicals in aqueous solution, with a slight superiority of GLA. Interestingly, GLA presents zero activity toward DPPH radicals. At the same time, material #2 exhibits a remarkable one, acting through a HAT mechanism involving the hydrogen atom of the hydroxyl group located near the sugar-O atom (Reaction 3).^21,58^ The anchoring of GLA on the silica surface to form the hybrid material #2 occurs via the carboxyl group, a process that highly increases its antioxidant activity.^21^
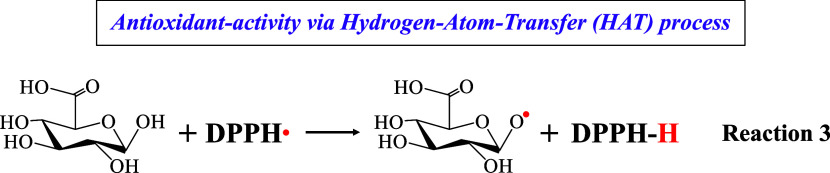


Concerning the activity of sugar GLA against hydroxyl (^•^OH) radicals, the HAT mechanism is employed again, which yields water and generates a free radical (Reaction 4), with the involvement of a loosely bound hydrogen atom on the α-carbon which bears the OH group,^54^ this OH which offers the hydrogen atom to quench the DPPH radical. It has been suggested that in aerobic environments, ^•^OH may initiate damage to polysaccharides through a sequence of reactions, i.e., ^•^OH abstracts a hydrogen atom bonded to a carbon atom, resulting in the formation of α-carbon-centered radical and water (H_2_O).^53^
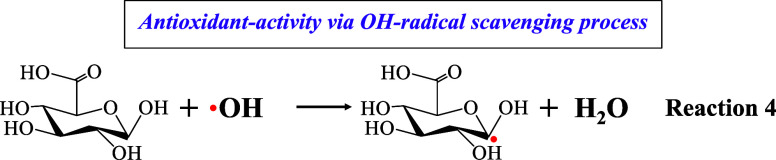


In our case, grafting of GLA on silica using the carboxylic group seems to result in a slight decrease of antioxidant activity in aqueous solution against ^•^OH vs free GLA. This could be attributed to the increased stability of GLA upon immobilization on the SiO_2_ surface, which is anticorrelated with a reduced rate of hydroxyl radical (^•^OH) reduction.^54^ Moreover, it was suggested by Morelli et al. that the mechanism of ^•^OH interaction might be related to the presence of stable free radical intermediates rather than solely attributed to sugar damage.^54^ That is, while it may seem initially that the antioxidant activity of GLA is diminished upon immobilization in silica, this perception is misleading since the formation of the hybrid material #2 stabilizes GLA, slightly reducing its reactivity with ^•^OH.

In the antioxidant activity of hybrid material #3, a significant difference exists concerning DPPH and hydroxyl (^•^OH) radicals. In the scavenging of the DPPH radicals via the HAT mechanism, the hydrogen atom of the hydroxyl group located near the sugar-O atom (Reaction 5) is expected to be involved;^58,59^ however, this site, being the most acidic one, has already been reacted during the GLAM grafting process on the silica surface.^21^ Thus, since this hydrogen atom is not more available, a very low antioxidant activity of material #3 is obtained against DPPH radicals. Analogous to GLA behavior, GLAM presents zero activity toward DPPH.
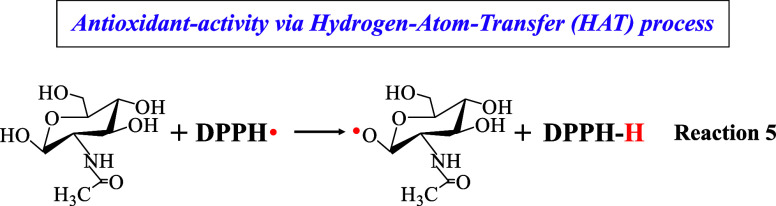


Consequently, GLAM and hybrid material #3 classified as sugars, exert their antioxidant activity against ^•^OH primarily through the HAT mechanism, whereas the abstracted hydrogen atom is derived from the α-carbon neutralizing them toward H_2_O (Reaction 6); in this process, GLAM itself is transformed into a radical.
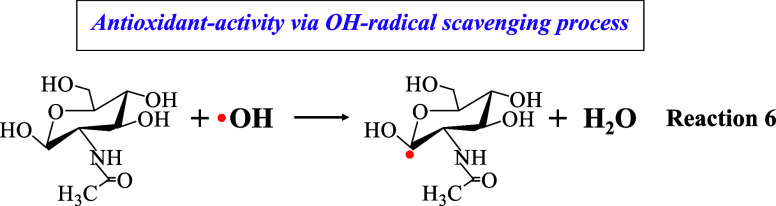


Material #3 disposes of this hydrogen atom for reaction with OH radicals; thus, HAT undergoes without freezing. Moreover, material #3 exhibits a greater capacity for scavenging ^•^OH compared to the free GLAM due to the hybrid material’s capability to regenerate by acquiring hydrogen atoms from the surrounding solvent, typically water. As a result, the hybrid material demonstrates enhanced stability and more effective quenching of ^•^OH.^54^

From the EPR data and the preceding discussion, it appears that the coimmobilization of the hyaluronic acid monomers GLA and GLAM on the same silica surface significantly enhances the material’s stability against free radicals. Consequently, the decrease in the EPR–OH signal intensity is less pronounced. In this context, we consider that the materials neutralize ^•^OH through the hydrogen atom transfer (HAT) mechanism, involving the detachment of a hydrogen atom from an α-carbon atom (Reactions 4 and 6). Due to its composition of two GLA molecules and one GLAM molecule, the doubly immobilized hybrid material #4 exhibits properties more akin to those of hybrid material #2 rather than #3. However, based on EPR signals, the antioxidant activity of the hybrid material #4 appears lower compared to that of the simply grafted #2 and #3 hybrids probably due to steric limitations caused by the high loading of GLA and GLAM on the silica surface. This is not the case for #4 in quenching the DPPH radicals, where the more exposed hydrogen atoms from the OH groups are employed.

**Figure 5:**
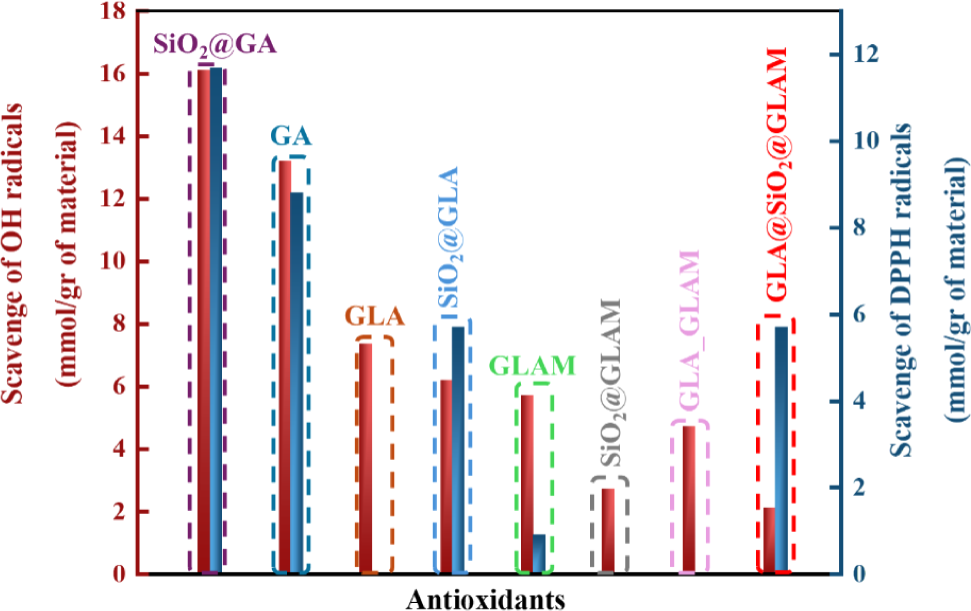
Ability to scavenge hydroxyl (^•^OH) and DPPH radicals, expressed in millimoles per gram of the respective material, is depicted above.

Finally, based on the data from Table 2 and Figure 5, hybrid material #1 exhibits the highest antioxidant activity of all materials, even from the free GA, highlighting the beneficial effect of GA grafting on its antioxidant efficacy. GA and material #1 neutralize hydroxyl (^•^OH) radicals through the single-electron transfer (SET) mechanism, where an electron is detached from a hydroxyl group of gallic acid. Contrarily, the silica-based hybrid materials #2 and #4 demonstrate a diminished capacity to scavenge ^•^OH, despite their increased stability, in comparison to their free counterparts. The sole hybrid material mirroring the behavior of material no. 1 is no. 3, wherein it displays enhanced antioxidant activity compared to free GLAM attributed to the ability of no. 3 to regenerate by acquiring a hydrogen atom from water. In general, the free molecules GLA, GLAM, and the simple mixture of GLA–GLAM display negligible antioxidant activity against DPPH radicals; however, concerning OH radicals, they exhibit a remarkably high antioxidant capacity. On the other hand, hybrid materials demonstrate significantly greater efficacy in scavenging DPPH radicals compared to their capability in binding hydroxyl (^•^OH) radicals (Figure 5).

The most notable finding among the aforementioned observations is the significant alteration in the behavior of the doubly grafted hybrid material no. 4, particularly regarding its capability to scavenge DPPH and hydroxyl (^•^OH) radicals. The doubly grafted material #4 demonstrates a superior capacity to scavenge DPPH radicals compared to the singly grafted hybrid materials #2 and #3. This suggests that, when both hyaluronic acid monomers are grafted on the same silica surface, they act synergistically, and, through the hydrogen atom transfer (HAT) mechanism, they effectively scavenge the DPPH radicals via the involvement of the H atom of the OH-group of GLA located nearby the sugar-O atom. The doubly grafted hybrid material #4 shows significantly lower antioxidant activity against ^•^OH compared to DPPH radicals; given that the adopted mechanism for quenching the ^•^OH is possibly the HAT via the C–H hydrogen atom, the observed low antioxidant activity is attributed to steric reasons due to the concomitant presence of grafted GLA and GLAM on SiO_2_, resulting in limited accessibility of C–H atoms (Figure 5).

Overall, the GA-based antioxidants operate via the single-electron transfer (SET) mechanism against ^•^OH and the hydrogen atom transfer (HAT) mechanism against DPPH radicals from the same OH atom of an OH group. On the other hand, the sugar-based antioxidants, GLA and GLAM, utilize a hydrogen-atom-transfer (HAT) mechanism against both hydroxyl (^•^OH) and DPPH radicals, with the involvement of a hydrogen atom of neighboring C–H and O–H groups, respectively. Taking into account, these mechanistic considerations with the adopted grafting process, which sets restrictions i.e., steric hindrance or blocking of specific sites, despite the stability it offers, can explain the observed antioxidant activity vs ^•^OH and DPPH radicals, of the antioxidants studied herein.

## 4 Conclusion

Using electron-paramagnetic resonance (EPR), we have studied the OH-radical scavenging efficiency of SiO_2_@GA, SiO_2_@GLA, SiO_2_@GLAM, and GLA@SiO_2_@GLAM (2:1). The data reveal that the most efficient OH-scavenger is SiO_2_@GA, which can neutralize 2.7 mol of hydroxyl radicals per mole of GA. Additionally, a comparative analysis of the capability of these materials to scavenge both DPPH^•^ and ^•^OH was conducted for the first time. The study demonstrated that all hybrid materials employ distinct mechanisms to interact with the two types of radicals. For OH-radicals, the mechanisms involved are single electron transfer (SET) and hydrogen atom transfer (HAT) from a carbon atom. In contrast, for DPPH radicals, the hydrogen atom transfer (HAT) mechanism involves hydrogen-atom abstraction from the OH groups of the organic moiety. Furthermore, in the double hybrid {GLA@SiO_2_@GLAM}, the two monomers synergistically enhance the antioxidant capacity against DPPH radicals but not the ability to neutralize hydroxyl (^•^OH) radicals. The ability of the hybrid material to scavenge ^•^OH is decreased due to steric hindrance affecting the carbon–hydrogen atoms caused by the presence of GLA and GLAM molecules.

## Abbreviations

GLA: d-glucuronic acid
GLAM: *N*-acetyl-d-glucosamine
GA: gallic acid
HAT: hydrogen atom transfer
SET: single electron transfer

## References

[ref1] Polak J.; Bartoszek M. The Study of Antioxidant Capacity of Varieties of Nalewka, a Traditional Polish Fruit Liqueur, Using EPR, NMR and UV–Vis Spectroscopy. J. Food Compos. Anal. 2015, 40, 114–119. 10.1016/j.jfca.2015.01.006.

[ref2] Hawkins C. L.; Davies M. J. Detection and Characterisation of Radicals in Biological Materials Using EPR Methodology. Biochim. Biophys. Acta, Gen. Subj. 2014, 1840 (2), 708–721. 10.1016/j.bbagen.2013.03.034. 23567797

[ref3] Suzen S.; Gurer-Orhan H.; Saso L. Detection of Reactive Oxygen and Nitrogen Species by Electron Paramagnetic Resonance (EPR) Technique. Molecules 2017, 22 (1), 181 10.3390/molecules22010181. 28117726 PMC6155876

[ref4] Fadda A.; Barberis A.; Sanna D. Influence of PH, Buffers and Role of Quinolinic Acid, a Novel Iron Chelating Agent, in the Determination of Hydroxyl Radical Scavenging Activity of Plant Extracts by Electron Paramagnetic Resonance (EPR). Food Chem. 2018, 240, 174–182. 10.1016/j.foodchem.2017.07.076. 28946259

[ref5] Valgimigli L.; Baschieri A.; Amorati R. Antioxidant Activity of Nanomaterials. J. Mater. Chem. B 2018, 6 (14), 2036–2051. 10.1039/C8TB00107C. 32254427

[ref6] Barroso-Martínez J. S.; Romo A. I. B.; Pudar S.; Putnam S. T.; Bustos E.; Rodríguez-López J. Real-Time Detection of Hydroxyl Radical Generated at Operating Electrodes via Redox-Active Adduct Formation Using Scanning Electrochemical Microscopy. J. Am. Chem. Soc. 2022, 144 (41), 18896–18907. 10.1021/jacs.2c06278. 36215201 PMC9586107

[ref7] Gao H. Y.; Huang C. H.; Mao L.; Shao B.; Shao J.; Yan Z. Y.; Tang M.; Zhu B. Z. First Direct and Unequivocal Electron Spin Resonance Spin-Trapping Evidence for PH-Dependent Production of Hydroxyl Radicals from Sulfate Radicals. Environ. Sci. Technol. 2020, 54 (21), 14046–14056. 10.1021/acs.est.0c04410. 33064470

[ref8] Shaeib F.; Banerjee J.; Maitra D.; Diamond M. P.; Abu-Soud H. M. Impact of Hydrogen Peroxide-Driven Fenton Reaction on Mouse Oocyte Quality. Free Radical Biol. Med. 2013, 58, 154–159. 10.1016/j.freeradbiomed.2012.12.007. 23261938 PMC4482232

[ref9] Burgos Castillo Rutely C.; Fontmorin J. -M.; Tang Walter Z.; Xochitl D. -B.; Mika S. Towards Reliable Quantification of Hydroxyl Radicals in the Fenton Reaction Using Chemical Probes. RSC Adv. 2018, 8 (10), 5321–5330. 10.1039/C7RA13209C. 35542446 PMC9078104

[ref10] Deng T.; Hu S.; Huang X. A.; Song J.; Xu Q.; Wang Y.; Liu F. A Novel Strategy for Colorimetric Detection of Hydroxyl Radicals Based on a Modified Griess Test. Talanta 2019, 195, 152–157. 10.1016/j.talanta.2018.11.044. 30625525

[ref11] Kukisawa T.; Kuwabara Y.; Nosaka A. Y.; Nosaka Y. Applications of Some EPR Methods to the Investigation of the Radical Species Produced by the Reactions of Hydroxyl Radicals with PEFC-Related Fluorinated Organic Acids. Phys. Chem. Chem. Phys. 2022, 24 (38), 23472–23480. 10.1039/D2CP02370A. 36128979

[ref12] Bartoszek M.; Polak J. An Electron Paramagnetic Resonance Study of Antioxidant Properties of Alcoholic Beverages. Food Chem. 2012, 132 (4), 2089–2093. 10.1016/j.foodchem.2011.12.060. 23871057

[ref13] Giordano A.; Morales-Tapia P.; Moncada-Basualto M.; Pozo-Martínez J.; Olea-Azar C.; Nesic A.; Cabrera-Barjas G. Polyphenolic Composition and Antioxidant Activity (ORAC, EPR and Cellular) of Different Extracts of Argylia Radiata Vitroplants and Natural Roots. Molecules 2022, 27 (3), 610 10.3390/molecules27030610. 35163871 PMC8838377

[ref14] Marchi R. C.; Campos I. A. S.; Santana V. T.; Carlos R. M. Chemical Implications and Considerations on Techniques Used to Assess the in Vitro Antioxidant Activity of Coordination Compounds. Coord. Chem. Rev. 2022, 451, 214275 10.1016/j.ccr.2021.214275.

[ref15] Davies M. J. Detection and Characterisation of Radicals Using Electron Paramagnetic Resonance (EPR) Spin Trapping and Related Methods. Methods 2016, 109 (2016), 21–30. 10.1016/j.ymeth.2016.05.013. 27211009

[ref16] Sanna D.; Fadda A. Role of the Hydroxyl Radical-Generating System in the Estimation of the Antioxidant Activity of Plant Extracts by Electron Paramagnetic Resonance (EPR). Molecules 2022, 27 (14), 4560 10.3390/molecules27144560. 35889433 PMC9316347

[ref17] Pei S.; You S.; Ma J.; Chen X.; Ren N. Electron Spin Resonance Evidence for Electro-Generated Hydroxyl Radicals. Environ. Sci. Technol. 2020, 54 (20), 13333–13343. 10.1021/acs.est.0c05287. 32931260

[ref18] Fontmorin J. M.; Burgos Castillo R. C.; Tang W. Z.; Sillanpää M. Stability of 5,5-Dimethyl-1-Pyrroline-N-Oxide as a Spin-Trap for Quantification of Hydroxyl Radicals in Processes Based on Fenton Reaction. Water Res. 2016, 99, 24–32. 10.1016/j.watres.2016.04.053. 27132196

[ref19] Khalil I.; Yehye W. A.; Etxeberria A. E.; Alhadi A. A.; Dezfooli S. M.; Julkapli N. B. M.; Basirun W. J.; Seyfoddin A. Nanoantioxidants: Recent Trends in Antioxidant Delivery Applications. Antioxidants 2019, 9 (1), 24 10.3390/antiox9010024. 31888023 PMC7022483

[ref20] Omran B.; Baek K. Nanoantioxidants: Pioneer Types, Advantages, Limitations, and Future Insights. Molecules 2021, 26 (22), 7031 10.3390/molecules26227031. 34834124 PMC8624789

[ref21] Theofanous A.; Sarli I.; Fragou F.; Bletsa E.; Deligiannakis Y.; Louloudi M. Antioxidant Hydrogen-Atom-Transfer to DPPH Radicals by Hybrids of {Hyaluronic-Acid Components}@SiO2. Langmuir 2022, 38 (40), 12333–12345. 10.1021/acs.langmuir.2c02021. 36165696

[ref22] Fragou F.; Theofanous A.; Deligiannakis Y.; Louloudi M. Nanoantioxidant Materials: Nanoengineering Inspired by Nature. Micromachines 2023, 14 (2), 383 10.3390/mi14020383. 36838085 PMC9963756

[ref23] Li X.; Li M.; Liu J.; Ji N.; Liang C.; Sun Q.; Xiong L. Preparation of Hollow Biopolymer Nanospheres Employing Starch Nanoparticle Templates for Enhancement of Phenolic Acid Antioxidant Activities. J. Agric. Food Chem. 2017, 65 (19), 3868–3882. 10.1021/acs.jafc.7b01172. 28467839

[ref24] Sudha A.; Jeyakanthan J.; Srinivasan P. Green Synthesis of Silver Nanoparticles Using Lippia Nodiflora Aerial Extract and Evaluation of Their Antioxidant, Antibacterial and Cytotoxic Effects. Resour. Technol. 2017, 3 (4), 506–515. 10.1016/j.reffit.2017.07.002.

[ref25] Medhe S.; Bansal P.; Srivastava M. M. Enhanced Antioxidant Activity of Gold Nanoparticle Embedded 3,6-Dihydroxyflavone: A Combinational Study. Appl. Nanosci. 2014, 4 (2), 153–161. 10.1007/s13204-012-0182-9.

[ref26] Ren J.; Li Q.; Dong F.; Feng Y.; Guo Z. Phenolic Antioxidants-Functionalized Quaternized Chitosan: Synthesis and Antioxidant Properties. Int. J. Biol. Macromol. 2013, 53, 77–81. 10.1016/j.ijbiomac.2012.11.011. 23164754

[ref27] Li Q.; Zhang C.; Tan W.; Gu G.; Guo Z. Novel Amino-Pyridine Functionalized Chitosan Quaternary Ammonium Derivatives: Design, Synthesis, and Antioxidant Activity. Molecules 2017, 22 (1), 156 10.3390/molecules22010156. 28106807 PMC6155944

[ref28] Magomedova E. F.; Pinyaskin V. V.; Aminova A. S. Synthesis and Antioxidant Activity of Azomethines. Pharm. Chem. J. 2007, 41 (9), 474–475. 10.1007/s11094-007-0104-4.

[ref29] Fa M.; Yang D.; Gao L.; Zhao R.; Luo Y.; Yao X. The Effect of AuNP Modification on the Antioxidant Activity of CeO 2 Nanomaterials with Different Morphologies. Appl. Surf. Sci. 2018, 457, 352–359. 10.1016/j.apsusc.2018.06.277.

[ref30] Lu M.; Zhang Y.; Wang Y.; Jiang M.; Yao X. Insight into Several Factors That Affect the Conversion between Antioxidant and Oxidant Activities of Nanoceria. ACS Appl. Mater. Interfaces 2016, 8 (36), 23580–23590. 10.1021/acsami.6b08219. 27548073

[ref31] Battaglini M.; Marino A.; Carmignani A.; Tapeinos C.; Cauda V.; Ancona A.; Garino N.; Vighetto V.; La Rosa G.; Sinibaldi E.; Ciofani G. Polydopamine Nanoparticles as an Organic and Biodegradable Multitasking Tool for Neuroprotection and Remote Neuronal Stimulation. ACS Appl. Mater. Interfaces 2020, 12 (32), 35782–35798. 10.1021/acsami.0c05497. 32693584 PMC8009471

[ref32] Qiu Y.; Wang Z.; Owens A. C. E.; Kulaots I.; Chen Y.; Kane A. B.; Hurt R. H. Antioxidant Chemistry of Graphene-Based Materials and Its Role in Oxidation Protection Technology. Nanoscale 2014, 6 (20), 11744–11755. 10.1039/C4NR03275F. 25157875 PMC4312421

[ref33] Merugu R.; Gothalwal R.; Kaushik Deshpande P.; De Mandal S.; Padala G.; Latha Chitturi K. Synthesis of Ag/Cu and Cu/Zn Bimetallic Nanoparticles Using Toddy Palm: Investigations of Their Antitumor, Antioxidant and Antibacterial Activities. Mater. Today Proc. 2021, 44, 99–105. 10.1016/j.matpr.2020.08.027.

[ref34] Sharma C.; Ansari S.; Ansari M. S.; Satsangee S. P.; Srivastava M. M. Single-Step Green Route Synthesis of Au/Ag Bimetallic Nanoparticles Using Clove Buds Extract: Enhancement in Antioxidant Bio-Efficacy and Catalytic Activity. Mater. Sci. Eng., C 2020, 116, 111153 10.1016/j.msec.2020.111153. 32806256

[ref35] Arriagada F.; Correa O.; Günther G.; Nonell S.; Mura F.; Olea-Azar C.; Morales J.; Xu B. Morin Flavonoid Adsorbed on Mesoporous Silica, a Novel Antioxidant Nanomaterial. PLoS One 2016, 11 (11), e0164507 10.1371/journal.pone.0164507. 27812111 PMC5094702

[ref36] Sandhya J.; Kalaiselvam S. Biogenic Synthesis of Magnetic Iron Oxide Nanoparticles Using Inedible Borassus Flabellifer Seed Coat: Characterization, Antimicrobial, Antioxidant Activity and in Vitro Cytotoxicity Analysis. Mater. Res. Express 2020, 7 (1), 015045 10.1088/2053-1591/ab6642.

[ref37] Sun Y.; Cui J.; Tian L.; Mi Y.; Guo Z. Phenolic Acid Functional Quaternized Chitooligosaccharide Derivatives: Preparation, Characterization, Antioxidant, Antibacterial, and Antifungal Activity. Mar. Drugs 2023, 21 (10), 535 10.3390/md21100535. 37888470 PMC10608605

[ref38] Pasanphan W.; Buettner G. R.; Chirachanchai S. Chitosan Gallate as a Novel Potential Polysaccharide Antioxidant: An EPR Study. Carbohydr. Res. 2010, 345 (1), 132–140. 10.1016/j.carres.2009.09.038. 19889400 PMC3695485

[ref39] Deligiannakis Y.; Sotiriou G. A.; Pratsinis S. E. Antioxidant and Antiradical SiO2 Nanoparticles Covalently Functionalized with Gallic Acid. ACS Appl. Mater. Interfaces 2012, 4 (12), 6609–6617. 10.1021/am301751s. 23121088

[ref40] Foti M. C.; Daquino C.; Mackie I. D.; DiLabio G. A.; Ingold K. U. Reaction of Phenols with the 2,2-Diphenyl-1-Picrylhydrazyl Radical. Kinetics and DFT Calculations Applied to Determine ArO-H Bond Dissociation Enthalpies and Reaction Mechanism. J. Org. Chem. 2008, 73 (23), 9270–9282. 10.1021/jo8016555. 18991378

[ref41] Foti M. C. Solvent Effects on the Activation Parameters of the Reaction between an Α-tocopherol Analogue and Dpph •: The Role of H-bonded Complexes. Int. J. Chem. Kinet. 2012, 44 (8), 524–531. 10.1002/kin.20619.

[ref42] Agarwal R. G.; Coste S. C.; Groff B. D.; Heuer A. M.; Noh H.; Parada G. A.; Wise C. F.; Nichols E. M.; Warren J. J.; Mayer J. M. Free Energies of Proton-Coupled Electron Transfer Reagents and Their Applications. Chem. Rev. 2022, 122 (1), 1–49. 10.1021/acs.chemrev.1c00521. 34928136 PMC9175307

[ref43] Fragou F.; Zindrou A.; Deligiannakis Y.; Louloudi M. Carbon-SiO_2_ Hybrid Nanoparticles with Enhanced Radical Stabilization and Biocide Activity. ACS Appl. Nano Mater. 2023, 6 (22), 20841–20854. 10.1021/acsanm.3c03852.

[ref44] Psathas P.; Moularas C.; Smykała S.; Deligiannakis Y. Highly Crystalline Nanosized NaTaO_3_ /NiO Heterojunctions Engineered by Double-Nozzle Flame Spray Pyrolysis for Solar-to-H 2 Conversion: Toward Industrial-Scale Synthesis. ACS Appl. Nano Mater. 2023, 6 (4), 2658–2671. 10.1021/acsanm.2c05066.

[ref45] Lloyd R. V.; Hanna P. M.; Mason R. P. The Origin of the Hydroxyl Radical Oxygenin the Fenton Reaction. Free Radical Biol. Med. 1997, 22 (5), 885–888. 10.1016/S0891-5849(96)00432-7. 9119257

[ref46] Kanigaridou Y.; Petala A.; Frontistis Z.; Antonopoulou M.; Solakidou M.; Konstantinou I.; Deligiannakis Y.; Mantzavinos D. I.; Kondarides D. I. Solar Photocatalytic Degradation of Bisphenol A with CuOx/BiVO4: Insights into the Unexpectedly Favorable Effect of Bicarbonates. Chem. Eng. J. 2017, 318, 39–49. 10.1016/j.cej.2016.04.145.

[ref47] Wang L.; Fu Y.; Li Q.; Wang Z. EPR Evidence for Mechanistic Diversity of Cu(II)/Peroxygen Oxidation Systems by Tracing the Origin of DMPO Spin Adducts. Environ. Sci. Technol. 2022, 56 (12), 8796–8806. 10.1021/acs.est.2c00459. 35608900

[ref48] Takayanagi T.; Kimiya H.; Ohyama T. Formation of Artifactual DMPO-OH Spin Adduct in Acid Solutions Containing Nitrite Ions. Free Radical Res. 2017, 51 (7–8), 739–748. 10.1080/10715762.2017.1369536. 28817986

[ref49] Bauer N. A.; Hoque E.; Wolf M.; Kleigrewe K.; Hofmann T. Detection of the Formyl Radical by EPR Spin-Trapping and Mass Spectrometry. Free Radical Biol. Med. 2018, 116, 129–133. 10.1016/j.freeradbiomed.2018.01.002. 29307725

[ref50] Fagan W. P.; Villamena F. A.; Zweier J. L.; Weavers L. K. In Situ EPR Spin Trapping and Competition Kinetics Demonstrate Temperature-Dependent Mechanisms of Synergistic Radical Production by Ultrasonically Activated Persulfate. Environ. Sci. Technol. 2022, 56 (6), 3729–3738. 10.1021/acs.est.1c08562. 35226467

[ref51] Walger E.; Marlin N.; Mortha G.; Molton F.; Duboc C. Hydroxyl Radical Generation by the H2o2/Cuii/Phenanthroline System under Both Neutral and Alkaline Conditions: An Epr/Spin-trapping Investigation. Appl. Sci. 2021, 11 (2), 687 10.3390/app11020687.

[ref52] Ueno M.; Nakanishi I.; Matsumoto K. I. Inhomogeneous Generation of Hydroxyl Radicals in Hydrogen Peroxide Solution Induced by Ultraviolet Irradiation and in a Fenton Reaction System. Free Radical Res. 2021, 55 (4), 481–489. 10.1080/10715762.2020.1819995. 32896187

[ref53] Hernandez-Marin E.; Martínez A. Carbohydrates and Their Free Radical Scavenging Capability: A Theoretical Study. J. Phys. Chem. B 2012, 116 (32), 9668–9675. 10.1021/jp304814r. 22853004

[ref54] Morelli R.; Russo-Volpe S.; Bruno N.; Lo Scalzo R. Fenton-Dependent Damage to Carbohydrates: Free Radical Scavenging Activity of Some Simple Sugars. J. Agric. Food Chem. 2003, 51 (25), 7418–7425. 10.1021/jf030172q. 14640593

[ref55] Marino T.; Galano A.; Russo N. Radical Scavenging Ability of Gallic Acid toward OH and OOH Radicals. Reaction Mechanism and Rate Constants from the Density Functional Theory. J. Phys. Chem. B 2014, 118 (35), 10380–10389. 10.1021/jp505589b. 25119432

[ref56] Pant A. F.; Özkasikci D.; Fürtauer S.; Reinelt M. Effect of Deprotonation on the Reaction Kinetics of an Oxygen Scavenger Based on Gallic Acid. Front. Chem. 2019, 7, 680 10.3389/fchem.2019.00680. 31781534 PMC6856669

[ref57] Llano S.; Gómez S.; Londoño J.; Restrepo A. Antioxidant Activity of Curcuminoids. Phys. Chem. Chem. Phys. 2019, 21 (7), 3752–3760. 10.1039/C8CP06708B. 30702098

[ref58] Parcheta M.; Swisłocka R.; Orzechowska S.; Akimowicz M.; Choińska R.; Lewandowski W. Recent Developments in Effective Antioxidants: The Structure and Antioxidant Properties. Materials 2021, 14, 1984 10.3390/ma14081984. 33921014 PMC8071393

[ref59] Samanta S.; Rangasami V. K.; Murugan N. A.; Parihar V. S.; Varghese O. P.; Oommen O. P. Polymer Chemistry on the Chemical Reactivity and Bioactivity Of.An unexpected role of an extra phenolic hydroxyl on the chemical reactivity and bioactivity of catechol or gallol modified hyaluronic acid hydrogels. Polym. Chem. 2021, 12, 2987–2991. 10.1039/D1PY00013F.

